# 

**Published:** 1890-07

**Authors:** 


### Southern Medical Record

A Monthly Journal of Medicine and Surgery.

VOL. XX. ATLANTA, GA., JULY, 1890. NO. 7.

EDITORS:

A. W. GRIGGS, M. D. FRANK O. STOCKTON, M. D.

WM. PERRIN NICOLSON, M D. DAN. H. HOWELL, M D., Bus. Mgr.

Entered at the Postoffice at Atlanta, Ga., as Second-Class Matter. Index to Advertisements on Page 18.

Postell & Sexton, Publishers, 47 S. Broad, Atlanta, Ga.

